# Empowered Relief, cognitive behavioral therapy, and health education for people with chronic pain: a comparison of outcomes at 6-month Follow-up for a randomized controlled trial

**DOI:** 10.1097/PR9.0000000000001116

**Published:** 2024-01-24

**Authors:** Beth D. Darnall, John W. Burns, Juliette Hong, Anuradha Roy, Kristin Slater, Heather Poupore-King, Maisa S. Ziadni, Dokyoung S. You, Corinne Jung, Karon F. Cook, Kate Lorig, Lu Tian, Sean C. Mackey

**Affiliations:** a Department of Anesthesiology, Perioperative and Pain Medicine, Stanford University School of Medicine, Palo Alto, CA, USA; b Department of Psychiatry and Behavioral Science, Rush University Medical Center, Chicago, IL, USA; c Feral Scholars, Broaddus, TX, USA; d Department of Rheumatology, Stanford University School of Medicine, Palo Alto, CA, USA; e Department of Biomedical Data Science and (by courtesy) Statistics, Stanford University School of Medicine, Palo Alto, CA, USA

**Keywords:** Chronic low back pain, Behavioral, CBT, Brief, Intervention, Treatment

## Abstract

**Introduction:**

We previously conducted a 3-arm randomized trial (263 adults with chronic low back pain) which compared group-based (1) single-session pain relief skills intervention (Empowered Relief; ER); (2) 8-session cognitive behavioral therapy (CBT) for chronic back pain; and (3) single-session health and back pain education class (HE). Results suggested non-inferiority of ER vs. CBT at 3 months post-treatment on an array of outcomes.

**Methods:**

Here, we tested the durability of treatment effects at 6 months post-treatment. We examined group differences in primary and secondary outcomes at 6 months and the degree to which outcomes eroded or improved from 3-month to 6-month within each treatment group.

**Results:**

Empowered Relief remained non-inferior to CBT on most outcomes, whereas both ER and CBT remained superior to HE on most outcomes. Outcome improvements within ER did not decrease significantly from 3-month to 6-month, and indeed ER showed additional 3- to 6-month improvements on pain catastrophizing, pain bothersomeness, and anxiety. Effects of ER at 6 months post-treatment (moderate term outcomes) kept pace with effects reported by participants who underwent 8-session CBT.

**Conclusions:**

The maintenance of these absolute levels implies strong stability of ER effects. Results extend to 6 months post-treatment previous findings documenting that ER and CBT exhibit similarly potent effects on outcomes.

## Abstract 2

Supplemental Digital Content is Available in the Text.

A One-session empowered relief provides clinically meaningful effects comparable to an 8-session cognitive behavioral therapy at 6 months for chronic low back pain. Effectiveness data are needed in diverse patients.

## 1. Introduction

Chronic low back pain is the most prevalent chronic pain condition among adults worldwide.^18^ Patient education on pain self-management and multisession cognitive behavioral therapy (CBT) are recommended as first-line treatments for back pain,^17^ and the National Institute for Health and Care Excellence (NICE) guidelines recommend CBT integration into comprehensive care plans.^27^ Often, group-based CBT for chronic pain is delivered over eight 2-hour sessions (16 hours total treatment time). Content includes pain education, self-regulatory skills training, problem-solving and action planning, and home practice between sessions. Rigorous back pain studies^8,38^ and chronic pain meta-analyses^41^ suggest CBT has small-to-moderate effects for multidimensional symptom reduction in chronic pain. However, multiple barriers prevent broad access to CBT, such as physician referral, lack of insurance in the U.S., lack of trained professionals, extensive wait times, and burdens associated with multisession treatment.^11,25^ Effective briefer options could ease care barriers, facilitate the implementation of recommended guidelines, and scale best practices to treat chronic low back pain.

Empowered Relief (ER) is a 2-hour single-session pain relief skills intervention that includes cognitive behavioral skills acquisition, mindfulness principles, and pain neuroscience education.^10,12,13^ Previously, we conducted a three-arm randomized controlled trial in 263 community adults with chronic low back pain in which we compared 2-hour ER, a 2-hour back health education (HE) class, and an 8-session back pain CBT protocol (16 hours of total treatment time).^7,8^ In this trial, pain catastrophizing, a cognitive and emotional pain response pattern that includes increased attention, and feelings of pain helplessness, was selected as the primary outcome because of its impacts on the intensity and trajectory of chronic pain^6,39^ and responsiveness to CBT.^38,40,41^ Results revealed noninferiority in outcome potency of a 2-hour pain relief skills intervention compared with a standard course of an 8-session CBT at 3 months posttreatment. Specifically, ER was noninferior to an 8-session CBT for reducing pain catastrophizing, pain intensity, pain interference, pain bothersomeness, fatigue, sleep disturbance, anxiety, and depression; across variables, ER and CBT had moderate-to-large treatment effects that were superior to HE at 3 months.^13^ Such findings suggest that for some patients, brief psychosocial pain interventions may represent satisfactory alternatives to more lengthy and resource-intensive treatment, at least in the short-term. Evidence of extended efficacy of 1-session ER could inform broad treatment adoption in clinical practice, resource allocation, and third-party payer reimbursement in the U.S.

Accordingly, the current report describes 6-month outcome data for our three-arm randomized comparative efficacy trial. First, we examined whether ER retained noninferiority to CBT and superiority to HE on baseline to 6-month posttreatment changes for primary and secondary outcomes. Results of these analyses also would indicate the relative position of an 8-session CBT and brief ER with respect to maintenance of longer-term effects. We hypothesized that at 6 months posttreatment, ER and CBT would be superior to HE and that ER would maintain noninferiority with CBT. Second, we examined the degree to which ER showed increments or decrements in outcome values from 3 months to 6 months posttreatment. Results of these analyses would indicate the degree to which effects of brief ER changed or maintained their *absolute* position from 3 months to 6 months posttreatment.

## 2. Methods

**Figure 1.**
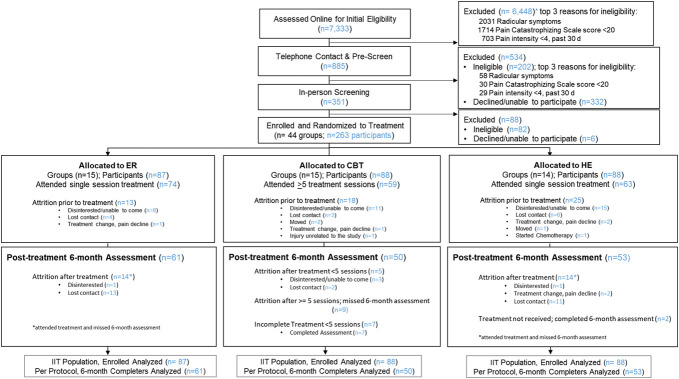
CONSORT diagram to 6 months posttreatment. CBT, cognitive behavioral therapy; ER, Empowered Relief; HE, health education.

We conducted this three-arm randomized comparative efficacy trial at a single academic site in the San Francisco Bay Area after trial preregistration^4^ and the published protocol.^12^ The trial tested for noninferiority in comparing a 1-session ER vs an 8-session CBT and superiority in comparing a 1-session ER vs a 1-session HE and an 8-session CBT vs a 1-session HE. The study protocol followed the Consolidated Standards of Reporting Trials (CONSORT) reporting guidelines on noninferiority trials (Fig. 1),^31^ was approved by Stanford's institutional review board, included a data and safety monitoring board, and the trial was overseen by an independent monitoring agency appointed by the National Institutes of Health.

### 2.1. Participants

We recruited participants from the community and clinics with advertisements for a no-cost, nondrug study involving 3 class-based treatments for chronic low back pain. Total compensation of USD $300 was possible for completing the study surveys.

We enrolled adults (aged 18–70 years) who met the NIH task force criteria for axial low back pain experienced on at least half of the days in the previous 6 months^14^ with an average pain intensity ≥4/10 and a Pain Catastrophizing Scale^34^ score ≥20 (moderate). Additional inclusion criteria were English fluency and the ability to attend up to eight 2-hour treatment sessions (in the event they were randomized to the CBT treatment group). Exclusion criteria included gross cognitive impairment, radicular symptoms, previous receipt of ER or CBT in the past 3 years, current substance use disorder, medicolegal factors, suicidal ideation, or severe depression (assessed with MINI 7.0 to screen,^33^ the Beck Depression Inventory-II for severity grading,^5^ and the Structured Clinical Interview for DSM-5 for diagnostics).^16^ Informed consent was obtained before enrollment.

### 2.2. Outcomes

Consistent with the published original NIH study protocol and CT.gov registration, the primary outcome for the noninferiority and superiority analyses was the 13-item Pain Catastrophizing Score,^34^ which measures the frequency of various cognitive or emotional responses to pain (eg, *There's nothing I can do to reduce the intensity of my pain*). Responses range from 0 (not at all) to 4 (all the time), and sum scores range from 0 to 52. The PCS is widely used in pain research and has good psychometric properties with clinic and community samples,^29,30^ including a high internal consistency (alpha = 0.87) and test-retest reliability (*r* = 0.75).^34^

Priority secondary outcomes included average pain intensity over the previous 7 days [range: 0 (no pain) to 10 (worst pain imaginable)]^15^ and PROMIS pain interference; other secondary outcomes included sleep disturbance, pain behavior, depression, anxiety, physical function, and fatigue (all evaluated with the National Institutes of Health [NIH] Patient-Reported Outcomes Measurement Information System short-form measures)^1,19^; pain bothersomeness during the previous 7 days [range: 0 (not at all bothersome) to 10 (extremely bothersome)]^8,38^; and pain self-efficacy via the Pain Self-Efficacy Questionnaire [range: 0 (not at all confident) to 6 (completely confident)].^28^

### 2.3. Randomization and investigator blinding

We randomly assigned participants to receive ER, HE, or CBT via Research Electronic Data Capture^20^ with no blocking applied. After randomization, individual treatment group allocations were revealed to participants and the study staff (project manager, study coordinators, and treatment instructors). All participants were told they were assigned to an active treatment for back pain, and HE participants were told they were receiving a back pain education class. The principal investigators, coinvestigators, and statisticians remained blinded to individual treatment group assignment until the three-month data were received. No efficacy analyses were performed before all 3-month data were collected.

Participant identification was protected with a unique study identification number. All data were received electronically, instantly locked in the database, and stored with double-password protection. Six-month posttreatment data collection was finalized in June 2020.

### 2.4. Study group interventions

We delivered treatments to small group cohorts of up to 12 participants in size for all 3 study groups. Treatment fidelity was assured by independent rater checklists that were completed during every treatment class. Study therapists delivered only one study intervention; study therapist intervention protocol training was commensurate across the 3 study treatment groups and all therapists.

#### 2.4.1. Empowered Relief

Empowered Relief is a 2-hour, 1-session intervention that includes pain neuroscience education, mindfulness principles, and pain and stress self-regulatory skills.^12,13,44^ Doctoral-level psychologists with 2 to 5 years of pain-specific clinical expertise were trained to deliver the manualized class using a standardized electronic slide deck and instructor manual. Participants received a binaural relaxation audio app and completed a personalized plan for Empowered Relief during the class. Ingredients of ER that overlap with CBT include elements of pain education, pain and stress self-regulatory skills, in-vivo experience of the relaxation response and provision of an ongoing relaxation audio tool, and completion of a personalized plan. Compared with CBT, key distinctions of ER include the structured didactic instructor slide deck presentation, content involving mindfulness principles, the binaural app, minimal participant sharing during the intervention, and lack of ongoing didactic content and peer and therapeutic support over the following 7 weeks.

#### 2.4.2. Back pain/health education

The HE class matched the ER class in duration, structure, format, and site; topics included warning signs for back pain, when to speak with your doctor, general nutrition, and pain medication management.^12^ A single instructor with 20 years of experience delivering health education interventions delivered the HE class using a standardized electronic slide deck. The HE intervention involved no actionable pain management skills or worksheets.

#### 2.4.3. Cognitive behavioral therapy for chronic low back pain

Doctoral-level psychologists with 2 to 25 years of pain-specific clinical expertise delivered eight 2-hour sessions of an established CBT protocol specific to chronic low back pain (16 hours total treatment time) to each treatment cohort.^7,8^ The content included a range of topics related to pain management (eg, mood, activity, sleep) and pain relief skills (eg, relaxation and cognitive restructuring). Participants received a workbook, 2 relaxation audio files, and a book for optional reading.^37^

### 2.5. Sample size and statistical analysis

A minimum sample size of 231 adults was needed to ensure adequate power to detect between group significant differences for changes in the primary outcome at 3 months posttreatment. The sample size accounted for predicted attrition rates of 25% attrition for the single-session classes and 35% attrition for an 8-session CBT. More details on the sample size are available in the study protocol.^12^

We summarized continuous and categorical baseline covariates using the mean (standard deviation) and count (proportions), respectively. To investigate the treatment effect at months 3 and 6, we applied mixed models for repeated measure (MMRM) regression to perform the intention-to-treat analysis.^13^ The dependent variable was the outcome of interest at baseline and months 1, 2, 3, and 6, and the independent variables were time (baseline, months 1, 2, 3, and 6; as categorical variables), treatment group (ER, CBT, HE; as categorical variables), and interactions between months and between treatment groups. We applied an unstructured covariance model to account for the within-subject correlations for outcomes of interest. The MMRM analysis allows missing at random assumption. Including outcomes at baseline, month 1, and month 2 increases the estimation precision for the treatment effect at months 3 and 6 in the presence of missing data. From the MMRM regression, we estimated the between-group differences and within-group differences in 3-month change from baseline, 6-month change from baseline, and change from month 3 to month 6. For the noninferiority analysis (ER vs CBT), one-sided confidence interval of the between group difference is constructed and compared with the specified noninferiority margin. For the primary outcome, pain catastrophizing score, we selected a noninferiority margin of 4.3, which was more stringent than the minimum clinically important difference (MCID) of 6.8 reported in the literature.^35^ Appendix 1, http://links.lww.com/PR9/A213 details the MCIDs selected for secondary study outcomes.^2,3,9,21–24,26,43^ Consistent with our previous publication of the 3-month posttreatment comparisons,^13^ we evaluated the noninferiority of ER to CBT based on a one-sided 97.5% confidence interval for pain catastrophizing score and other endpoints, except for pain intensity and interference scores (priority secondary endpoints), whose noninferiority was based on one-sided 98.75% confidence interval to adjust for 2 comparisons. For corresponding superiority of CBT to HE and ER to HE, 2-sided 95% confidence interval was used for all endpoints, except for 2 priority secondary endpoints, which are based on 2-sided 97.5% confidence interval. The effect sizes for average improvement for all outcomes were calculated using Glass' δ, with the absolute mean change from baseline to month 3 or month 6 divided by the standard deviation at baseline.

To examine the missing pattern, we summarized the attrition rate by treatment group at each stage and compared baseline characteristics between those who completed posttreatment surveys and those who did not. We performed analyses using SAS Enterprise Guide version 8.3 (SAS Institute Inc, Cary, NC).

## 3. Results

### 3.1. Participants

The study sample (N = 263) was predominantly white (60.2%), non-Hispanic (93.4%), married/cohabitating (60.8%), with at least some college education (97.7%). About 64.6% of the sample reported having low back pain for at least 5 years, and almost half of the sample (48%) reported having at least one comorbid chronic pain condition. Although rates of formally diagnosed depressive mood disorder and anxiety disorder (using the MINI 7.0 and the Structured Clinical Interview for *DSM-5* Disorders) were relatively low, baseline elevated symptom levels for PROMIS depression and anxiety aligned with other studies on patients seeking treatment in tertiary pain clinics.^32,45^ Table 1 displays additional clinical characteristics and treatment history data; while we report these data for completeness in the sample description, details regarding baseline measures (eg, treatment expectancy measures) are discussed in the previous report.^13^

**Table 1:** Baseline demographic and clinical characteristics by treatment group (intention-to-treat).

Characteristic	No. (%)			
	Total sample (n = 263)	Empowered Relief (n = 87)	Cognitive behavioral therapy (n = 88)	Health education (n = 88)
Age, mean (SD), y	47.9 (13.8)	49.7 (15.0)	45.9 (13.1)	48.0 (13.2)
Sex				
Female	131 (49.8)	44 (50.6)	40 (45.5)	47 (53.4)
Male	130 (49.4)	42 (48.3)	47 (53.4)	41 (46.6)
Other	2 (0.8)	1 (1.1)	1 (1.1)	0 (0.0)
Race				
White	157 (60.2)	57 (66.3)	48 (54.5)	52 (59.8)
Asian/Pacific Islander	64 (24.5)	16 (18.6)	27 (30.7)	21 (24.1)
African American	11 (4.2)	5 (5.8)	4 (4.5)	2 (2.3)
American Indian/Alaska Native	2 (0.8)	1 (1.2)	0 (0.0)	1 (1.1)
Other	27 (10.3)	7 (8.1)	9 (10.2)	11 (12.6)
Ethnicity				
Hispanic	17 (6.6)	7 (8.3)	6 (6.9)	4 (4.6)
Non-Hispanic	241 (93.4)	77 (91.7)	81 (93.1)	83 (95.4)
Relationship status				
Married/cohabitating	160 (60.8)	53 (60.9)	58 (65.9)	49 (55.7)
Never married	71 (27.0)	27 (31.0)	22 (25.0)	22 (25.0)
Divorced	22 (8.4)	5 (5.7)	4 (4.5)	13 (14.8)
Separated	6 (2.3)	1 (1.1)	1 (1.1)	4 (4.5)
Widowed	4 (1.5)	1 (1.1)	3 (3.4)	0 (0.0)
Education				
High school	6 (2.3)	2 (2.3)	2 (2.3)	2 (2.3)
Some college	66 (25.1)	23 (26.4)	17 (19.3)	26 (29.5)
Bachelor's degree	90 (34.2)	30 (34.5)	32 (36.4)	28 (31.8)
Master's degree	67 (25.5)	21 (24.1)	26 (29.5)	20 (22.7)
Doctoral degree	34 (12.9)	11 (12.6)	11 (12.5)	12 (13.6)
Employment				
Full-time	114 (43.3)	30 (34.5)	45 (51.1)	39 (44.3)
Part-time	49 (18.6)	17 (19.5)	14 (15.9)	18 (20.5)
Retired	39 (14.8)	22 (25.3)	9 (10.2)	8 (9.1)
Student	16 (6.1)	6 (6.9)	6 (6.8)	4 (4.5)
Unemployed	16 (6.1)	4 (4.6)	6 (6.8)	6 (6.8)
Disabled	13 (4.9)	4 (4.6)	4 (4.5)	5 (5.7)
Household income, $USD				
<30,000	31 (12.3)	14 (16.5)	7 (8.5)	10 (11.6)
<50,000	27 (10.7)	9 (10.6)	7 (8.5)	11 (12.8)
<70,000	30 (11.9)	12 (14.1)	10 (12.2)	8 (9.3)
≥70,000	165 (65.2)	50 (58.8)	58 (70.7)	57 (66.3)
Smoking status				
Never smoked	175 (66.8)	58 (66.7)	65 (73.9)	52 (59.8)
Current	20 (7.6)	5 (5.7)	6 (6.8)	9 (10.3)
Past	67 (25.6)	24 (27.6)	17 (19.3)	26 (29.9)
BMI, mean (SD), kg/m^2^	27.0 (6.3)	27.3 (6.0)	27.0 (6.5)	26.7 (6.3)
Pain duration				
6–12 mo	14 (5.3)	4 (4.6)	8 (9.1)	2 (2.3)
1–5 y	79 (30.0)	25 (28.7)	24 (27.3)	30 (34.1)
>5 y	170 (64.6)	58 (66.7)	56 (63.6)	56 (63.6)
Back pain intensity, mean (SD)				
Past 30 d	5.8 (1.3)	5.6 (1.3)	5.9 (1.3)	6.0 (1.3)
Treatment expectations, mean (SD)*				
Positive	3.69 (1.27)	3.71 (1.30)	3.74 (1.22)	3.60 (1.32)
Negative	2.14 (1.30)	2.29 (1.34)	2.00 (1.12)	2.12 (1.43)
Comorbid pain condition†				
1	127 (48.3)	38 (43.7)	43 (48.9)	46 (52.3)
2+	48 (18.3)	18 (20.7)	15 (17.0)	15 (17.0)
Fibromyalgia	10 (3.8)	2 (2.3)	4 (4.5)	4 (4.5)
Complex regional pain syndrome	3 (1.1)	1 (1.1)	0 (0.0)	2 (2.3)
Pelvic pain	22 (8.4)	4 (4.6)	9 (10.2)	9 (10.2)
Migraine	31 (11.8)	13 (14.9)	8 (9.1)	10 (11.4)
Pain, other	160 (60.8)	54 (62.1)	54 (61.4)	52 (59.1)
Medication use for CLBP				
Opioids	43 (16.3)	13 (14.9)	17 (19.3)	13 (14.8)
NSAID/acetaminophen‡	123 (46.8)	45 (51.7)	38 (43.2)	40 (45.5)
Adjunctive pain medications§	66 (25.1)	22 (25.3)	29 (33.0)	15 (17.0)
Mental health disorders				
Mood disorders				
Ever	132 (50.2)	44 (50.6)	44 (50.0)	44 (50.0)
Current	16 (6.1)	9 (10.3)	4 (4.5)	3 (3.4)
Past	131 (49.8)	43 (49.4)	44 (50.0)	44 (50.0)
Anxiety disorders	82 (31.2)	24 (27.6)	33 (37.5)	25 (28.4)

Wald χ^2^ test used for categorical variables; F-test used for continuous variables, *P* values all non-significant between groups.

* Stanford Expectations of Treatment Scale (SETS).

† Pain conditions comorbid with chronic low back pain, excluding post-surgical pain.

‡ Prescription and/or over-the-counter.

§ Neuropathic pain medication, muscle relaxant, and all other pain related medication (OTC or prescription).

BMI, body mass index; CLBP, chronic low back pain; OTC, over-the-counter.

Figure 1 provides the CONSORT diagram and participant flow of this study. Among participants who completed their assigned treatment, the attrition rate at 6 months posttreatment was as follows: ER, 19% (14 of 74); CBT, 15% (9 of 59); and HE, 22% (14 of 63). No adverse events related to the study were reported during the study period. No differences in sociodemographic or clinical characteristics were found between those who completed the 6-month follow-up survey and those that did not.

### 3.2. Comparisons of treatment groups at 6 months posttreatment

Table 2 displays the raw means by time point, and Table 3 displays the between-group comparisons of treatment effects at 3 and 6 months posttreatment. In general, the similarity of effects at 3-months for ER vs CBT were maintained at 6 months posttreatment, with a few exceptions. That is, ER was noninferior to CBT for reducing the pain catastrophizing scale (primary outcome), pain intensity (priority secondary outcomes), self-efficacy, pain bothersomeness, pain behavior, fatigue, depression, and anxiety at 3 and 6 months posttreatment. Empowered Relief was noninferior to CBT for reducing sleep disturbance at 3 months posttreatment only. Empowered Relief was noninferior to CBT for reducing pain interference (priority secondary outcome), and physical function at 6 months posttreatment only. Furthermore, at 6 months posttreatment, the ER group had statistically significantly greater reductions in pain catastrophizing (primary outcome) compared with CBT (−3.62; 97.5% CI −∞ to −0.30; *P* = 0.03). Empowered Relief also showed greater reductions in pain bothersomeness (−0.98; 97.5% CI −∞ to −0.16; *P* = 0.02) and anxiety (−3.32; 97.5% CI −∞ to −0.16; *P* = 0.04) compared with CBT at 6 months posttreatment.

**Table 2:** Outcome measures with mean (SD) over time by treatment group.

Outcome measure	Empowered Relief		Cognitive behavioral therapy		Health education	
Time point	N	Mean (SD)	N	Mean (SD)	N	Mean (SD)
Pain catastrophizing						
Baseline	78	22.09 (9.84)	76	23.01 (8.98)	69	24.81 (10.32)
3 mo	64	13.17 (10.15)	61	11.87 (9.25)	58	19.74 (9.95)
6 mo	61	10.98 (9.95)	50	14.66 (10.24)	53	17.74 (10.81)
Pain intensity						
Baseline	76	4.16 (1.73)	74	4.96 (1.68)	68	4.93 (1.59)
3 mo	63	3.14 (2.02)	60	3.20 (2.07)	56	4.41 (1.97)
6 mo	60	2.82 (1.87)	50	3.46 (2.51)	53	4.43 (1.80)
PROMIS pain interference						
Baseline	77	58.33 (6.45)	75	61.61 (6.06)	68	60.83 (5.17)
3 mo	63	54.06 (8.34)	60	53.89 (8.65)	57	58.85 (6.67)
6 mo	60	53.32 (7.07)	50	55.41 (9.07)	53	58.83 (6.84)
PROMIS sleep disturbance						
Baseline	77	55.13 (8.22)	75	56.20 (7.33)	67	57.04 (6.75)
3 mo	63	50.01 (9.20)	60	52.65 (9.76)	57	57.14 (7.92)
6 mo	60	50.83 (9.64)	50	52.13 (8.07)	53	54.99 (7.78)
Pain self-efficacy (PSEQ)						
Baseline	77	39.26 (11.99)	76	35.25 (11.33)	68	35.49 (11.55)
3 mo	63	44.54 (11.73)	60	45.35 (13.18)	57	37.91 (12.10)
6 mo	61	45.92 (12.19)	50	44.82 (12.42)	53	38.40 (13.23)
Pain bothersomeness						
Baseline	76	4.58 (2.11)	75	5.95 (2.25)	67	5.69 (1.93)
3 mo	63	3.30 (2.33)	60	3.40 (2.57)	57	4.86 (2.36)
6 mo	60	3.07 (2.24)	50	4.02 (2.92)	52	5.06 (1.97)
PROMIS pain behavior						
Baseline	77	58.97 (3.43)	75	59.22 (4.15)	68	59.59 (2.87)
3 mo	63	54.58 (6.70)	60	55.61 (6.55)	57	58.35 (3.50)
6 mo	60	54.05 (6.77)	50	55.52 (7.41)	53	58.25 (3.35)
PROMIS fatigue						
Baseline	77	57.60 (8.05)	75	60.46 (8.79)	68	59.09 (6.96)
3 mo	63	53.43 (10.93)	60	53.01 (11.37)	57	56.63 (7.44)
6 mo	60	51.64 (11.28)	50	54.42 (10.84)	53	56.41 (6.95)
PROMIS depression						
Baseline	77	53.18 (9.11)	75	55.52 (7.88)	67	55.23 (8.49)
3 mo	63	49.93 (9.41)	60	52.11 (8.85)	57	54.56 (9.04)
6 mo	60	49.05 (8.91)	50	52.37 (10.03)	52	53.52 (10.20)
PROMIS anxiety						
Baseline	77	54.95 (9.85)	75	57.41 (7.42)	67	55.51 (8.76)
3 mo	63	51.09 (9.94)	60	52.89 (10.06)	57	54.82 (9.49)
6 mo	60	49.87 (10.11)	50	54.12 (9.63)	52	54.05 (9.78)
PROMIS physical function						
Baseline	77	42.63 (6.17)	75	40.53 (6.09)	67	40.72 (5.39)
3 mo	63	44.45 (7.93)	60	45.13 (7.59)	57	41.36 (5.96)
6 mo	60	45.73 (7.58)	50	43.92 (9.03)	52	42.07 (7.34)
Chronic pain acceptance						
Baseline	77	31.66 (5.28)	76	28.93 (5.58)	68	29.31 (6.04)
3 mo	64	32.20 (6.10)	61	28.87 (5.28)	57	28.91 (5.19)
6 mo	61	33.56 (6.36)	50	30.12 (6.39)	53	29.55 (4.96)

PROMIS, patient-reported outcomes measurement information system.

**Table 3:** Posttreatment months 3 to 6 by treatment group with between-group comparisons (intent-to-treat).

Outcome measure time point	Cognitive behavioral therapy vs health education		Empowered Relief vs health education		Empowered Relief vs cognitive behavioral therapy	
	Difference (CI*)	*P*	Difference (CI*)	*P*	Difference (CI†)	*P*
Pain Catastrophizing Scale‡						
3 mo	−8.19 (−11.5, −4.88)	<0.001§^CBT^	−7.43 (−10.7, −4.15)	<0.001§^ER^	0.76 (-∞, 3.99)	0.64
6 mo	−4.22 (−7.65, −0.79)	0.02‖^CBT^	−7.85 (−11.2, −4.51)	<0.001§^ER^	−3.62 (-∞,-0.30)	0.03‖^ER^
Pain Intensity#						
3 mo	−1.07 (−2.34, 0.19)**	0.39	−1.34 (−2.60, −0.09)**	0.02‖^ER^	−0.27 (-∞, 0.96)††	1.00
6 mo	−1.03 (−2.33, 0.28)**	0.81	−1.72 (−2.99, −0.45)**	<0.001§^ER^	−0.70 (-∞, 0.58)††	1.00
PROMIS pain interference#						
3 mo	−4.37 (−8.99, 0.25)**	0.11	−4.75 (−9.34, −0.17)**	0.03‖^ER^	−0.38 (-∞, 4.12)††	1.00
6 mo	−3.84 (−8.60, 0.93)**	0.64	−5.50 (−10.1, −0.85)**	0.003‖^ER^	−1.66 (-∞, 2.97)††	1.00
PROMIS sleep disturbance						
3 mo	−4.80 (−7.65, −1.94)	0.001‖^CBT^	−6.90 (−9.72, −4.07)	<0.001§^ER^	−2.10 (-∞, 0.67)	0.14
6 mo	−3.08 (−6.03, −0.13)	0.04‖^CBT^	−4.00 (−6.87, −1.12)	0.006‖^ER^	−0.92 (-∞, 1.95)	0.53
Pain self efficacy						
3 mo	6.53 (2.43, 10.62)	0.002‖^CBT^	6.63 (2.56, 10.69)	0.001‖^ER^	0.10 (−3.88, ∞)	0.96
6 mo	6.45 (2.26, 10.63)	0.003‖^CBT^	7.94 (3.84, 12.04)	<0.001§^ER^	1.49 (−2.57, ∞)	0.47
Pain bothersomeness						
3 mo	−1.31 (−2.12, −0.51)	0.001‖^CBT^	−1.62 (−2.43, −0.82)	<0.001§^ER^	−0.31 (-∞, 0.48)	0.44
6 mo	−1.05 (−1.89, −0.21)	0.01‖^CBT^	−2.03 (−2.85, −1.21)	<0.001§^ER^	−0.98 (-∞, −0.16)	0.02‖^ER^
PROMIS pain behavior						
3 mo	−2.44 (−4.25, −0.63)	0.008‖^CBT^	−3.46 (−5.25, −1.67)	<0.001§^ER^	−1.02 (-∞, 0.74)	0.26
6 mo	−3.14 (−5.02, −1.26)	0.001‖ ^CBT^	−4.14 (−5.96, −2.32)	<0.001§^ER^	−1.00 (-∞, 0.82)	0.28
PROMIS fatigue						
3 mo	−3.23 (−6.33, −0.13)	0.04‖ ^CBT^	−3.16 (−6.23, −0.09)	0.04‖^ER^	0.07 (-∞, 3.09)	0.96
6 mo	−2.82 (−6.02, 0.37)	0.08	−4.51 (−7.63, −1.40)	0.005‖^ER^	−1.69 (-∞, 1.42)	0.29
PROMIS depression						
3 mo	−2.36 (−5.50, 0.77)	0.14	−4.31 (−7.43, −1.20)	0.007‖^ER^	−1.95 (-∞, 1.10)	0.21
6 mo	−1.10 (−4.32, 2.11)	0.50	−3.75 (−6.91, −0.60)	0.02‖^ER^	−2.65 (-∞, 0.46)	0.10
PROMIS anxiety						
3 mo	−1.83 (−5.02, 1.35)	0.26	−3.83 (−7.00, −0.67)	0.02‖^ER^	−2.00 (-∞, 1.10)	0.21
6 mo	−0.69 (−3.96, 2.58)	0.68	−4.01 (−7.22, −0.81)	0.01‖^ER^	−3.32 (-∞, −0.16)	0.04‖^ER^
PROMIS physical function						
3 mo	2.97 (0.64, 5.30)	0.01‖^CBT^	3.21 (0.90, 5.53)	0.006‖^ER^	0.24 (−2.01, ∞)	0.83
6 mo	2.63 (0.26, 5.01)	0.03‖^CBT^	4.11 (1.77, 6.44)	0.001‖^ER^	1.48 (−0.82, ∞)	0.21
Chronic pain acceptance						
3 mo	−0.09 (−2.09, 1.90)	0.93	3.36 (1.39, 5.34)	0.001‖^ER^	3.45 (1.52, ∞)	<0.001§^ER^
6 mo	0.81 (−1.26, 2.89)	0.44	4.36 (2.35, 6.37)	<0.001§^ER^	3.54 (1.53, ∞)	0.001‖^ER^

* Two-sided 95% confidence interval.

† One-sided 97.5% confidence interval.

‡ Primary outcome.

§ *P*-value <0.001; All *P* values are based on 2-sided confidence interval.

‖ *P*-value <0.05.

# Priority secondary outcome; Bonferroni adjustment is applied for confidence interval and *P* value.

** Two-sided 97.5% confidence interval.

†† One-sided 98.75% confidence interval.

CI, confidence interval; PROMIS, patient-reported outcomes measurement information system.

The superiority of effects of ER and CBT vs HE at 3 months posttreatment was maintained, again with few exceptions, at 6 months posttreatment. Specifically, at 6 months posttreatment, ER remained superior to HE for reducing pain intensity (−1.72; 97.5% CI −2.99 to −0.45; *P* < 0.001) and pain interference (−5.50; 97.5% CI −10.1 to −0.85; *P* = 0.003); however, for CBT vs HE, these comparative effects are not statistically significant.

### 3.3. Within treatment group comparisons of 3-month to 6-month changes

Table 4 displays the differences in outcomes between 3 months and 6 months posttreatment for ER, CBT, and HE separately. For HE, all baseline to 3-month gains—albeit smaller in magnitude than for ER and CBT—were maintained at 6 months without exception. For CBT, all baseline to 3-month posttreatment gains were maintained at 6 months posttreatment with the exception of pain catastrophizing. In this case, pain catastrophizing scores significantly increased from 3 months to 6 months, signaling a slight decay of previous improvements for this pain-related cognition. For ER, maintenance of 3-month gains at 6 months was also typical, but with 3 noteworthy exceptions. It should be noted that in our study describing baseline to 3-month outcomes, we reported inferiority of ER to CBT for physical function; however, a scoring error in RedCap was later discovered in which one item in the item bank was not scored. Once this error was corrected, results supported noninferiority for ER vs CBT for physical function at 3 months posttreatment. This scoring error was corrected for all groups and across all time points in the current report. For ER, from 3 to 6 months posttreatment, physical function, pain acceptance, and pain catastrophizing significantly improved (ie, physical function and pain acceptance scores increased, pain catastrophizing scores decreased).

**Table 4:** Within-group changes from 3 months posttreatment to 6 months posttreatment.

Outcome	Empowered Relief		Cognitive behavioral therapy		Health education	
	Difference (CI*)	*P*	Difference (CI*)	*P*	Difference (CI*)	*P*
Pain catastrophizing scale	−2.06 (−3.90, −0.22)	0.03†	2.31 (0.26, 4.35)	0.03†	−1.47 (−3.47, 0.52)	0.15
Pain intensity	−0.25 (−0.64, 0.14)	0.21	0.19 (−0.25, 0.62)	0.40	0.14 (−0.29, 0.57)	0.53
PROMIS pain interference	−0.54 (−1.96, 0.88)	0.45	0.90 (−0.68, 2.48)	0.26	0.43 (−1.11, 1.98)	0.58
Pain bothersomeness	−0.15 (−0.72, 0.42)	0.60	0.57 (−0.06, 1.20)	0.08	0.28 (−0.34, 0.90)	0.37
PROMIS sleep disturbance	0.77 (−1.24, 2.79)	0.45	−0.56 (−2.78, 1.66)	0.62	−1.82 (−4.00, 0.35)	0.10
PROMIS pain behavior	−0.45 (−1.60, 0.69)	0.43	−0.57 (−1.84, 0.70)	0.38	0.12 (−1.13, 1.36)	0.85
PROMIS fatigue	−1.53 (−3.62, 0.56)	0.15	0.29 (−2.02, 2.60)	0.81	0.03 (−2.23, 2.30)	0.98
PROMIS depression	−0.62 (−2.31, 1.07)	0.47	0.07 (−1.81, 1.95)	0.94	−1.21 (−3.06, 0.65)	0.20
PROMIS anxiety	−0.81 (−2.52, 0.91)	0.35	0.24 (−1.67, 2.16)	0.80	−0.90 (−2.78, 0.98)	0.35
PROMIS physical function	1.43 (0.31, 2.56)	0.01†	0.27 (−0.99, 1.52)	0.68	0.21 (−1.02, 1.45)	0.73
Chronic pain acceptance	1.38 (0.05, 2.71)	0.04†	1.12 (−0.34, 2.58)	0.13	0.50 (−0.95, 1.94)	0.50
Pain self efficacy	1.04 (−0.97, 3.05)	0.31	0.48 (−1.77, 2.73)	0.67	−0.21 (−2.40, 1.98)	0.85

* Two-sided 95% confidence interval.

† Statistical significance: *P*-value <0.05.

CI, confidence interval; PROMIS, patient-reported outcomes measurement information system.

## 4. Discussion

Our study evaluated the relative and absolute durability of the treatment efficacy of a 1-session intervention (ER) compared with an 8-session CBT for adults with chronic lower back pain. In general, 6 months posttreatment results suggest that participants randomized to the 1-session ER maintained gains relative to the gains shown by participants randomized to the full course of CBT. That is, for the most part, effects of ER at 6 months posttreatment kept pace with effects reported by participants who underwent the 8-session CBT. Moreover, results indicate that 6-month levels of outcomes for ER did not significantly decline from gains shown at 3 months. The maintenance of these absolute levels implies strong stability of ER effects. Results extend previous findings documenting that ER and CBT exhibit similarly potent effects on outcomes^13^; here, out to 6 months posttreatment. In addition, the average improvement at 3 and 6 months yielded large treatment effect sizes for ER and CBT for reducing pain catastrophizing and pain intensity, and for CBT only, also pain interference (Glass' δ reported in Table 5). Effect sizes were moderate for all other variables for CBT and ER. Compared with minimally important differences (MID), the 6-month improvement in pain interference among patients from the ER group surpassed the preregistered MID (5.01 vs 4.0 MID), but the corresponding improvement in pain intensity fell short of the MID of pain intensity (1.34 vs 1.5 MID); the 6-month improvement in CBT group surpassed the MID for pain intensity (1.5 vs 1.5 MID) and pain interference (6.2 vs 4.0 MID). However, in comparison with HE, although the treatment benefits of ER in pain intensity and pain interference are statistically significant at the 0.05 level, their confidence intervals include the MID and we cannot conclude that the benefits are greater than the MID.

**Table 5:** Effect size* at post-treatment month 3 and month 6

Time	Variable	Empowered Relief	Cognitive behavioral therapy	Health education
Month 3 post-treatment				
	Pain Catastrophizing Scale	0.93	1.24	0.41
	Pain intensity	0.64	0.96	0.29
	PROMIS pain interference	0.64	1.12	0.36
	Pain bothersomeness	0.64	1.02	0.40
	PROMIS sleep disturbance	0.54	0.47	0.08
	PROMIS fatigue	0.45	0.73	0.33
	PROMIS depression	0.31	0.41	0.10
	PROMIS anxiety	0.35	0.52	0.03
	PROMIS physical function	0.41	0.62	0.19
	PROMIS pain behavior	1.26	0.74	0.45
	Pain self efficacy	0.43	0.85	0.17
	Chronic pain acceptance	0.15	0.01	0.04
Month 6 post-treatment				
	Pain Catastrophizing Scale	1.14	1.06	0.63
	Pain intensity	0.80	0.86	0.31
	PROMIS pain interference	0.71	0.96	0.33
	Pain bothersomeness	0.71	0.77	0.25
	PROMIS sleep disturbance	0.41	0.40	0.21
	PROMIS fatigue	0.65	0.69	0.38
	PROMIS depression	0.38	0.43	0.25
	PROMIS anxiety	0.44	0.44	0.07
	PROMIS physical function	0.62	0.67	0.34
	PROMIS pain behavior	1.40	0.86	0.43
	Pain self efficacy	0.54	0.85	0.27
	Chronic pain acceptance	0.39	0.17	0.00

* Effect size calculated by Glass' δ, difference of mean divided by standard deviation, where difference is of the paired samples values.

PROMIS, patient-reported outcomes measurement information system.

The noninferiority of ER compared with CBT on primary and secondary outcomes at 3 months posttreatment was largely sustained at 6 months posttreatment. Empowered Relief remained noninferior compared with CBT for pain intensity and pain interference, sleep disturbance, pain self-efficacy, pain behavior, fatigue, and depressive symptoms. In addition, at 6 months posttreatment, ER showed noninferiority to CBT for physical function, an effect not reported at 3 months because of the aforementioned scoring issue.^13^ Findings also suggested that ER was superior to CBT at 6 months for reducing pain catastrophizing, pain bothersomeness, and anxiety. Finally, ER effects on outcomes at 6 months remained superior to those of HE. Results suggest that the effects of ER, moving from 3 months to 6 months posttreatment, maintained their positions relative to the effects shown by CBT and HE during this period. However, between-group positions of effects for each treatment would be stable even where all effects decayed at the same rate.

To address the possibility of similar rates of decay, further analyses focused on within treatment group changes in outcome values from 3 months to 6 months posttreatment. Results showed that all outcome gains for ER and most effects for CBT did not reveal retreats from 3-month gains over this period. Indeed, compared with their 3-month levels, participants in ER reported increases in physical function and pain acceptance and decreases in pain catastrophizing at 6 months posttreatment. Results suggest that the absolute levels of outcome gains achieved at 3 months posttreatment were maintained or even amplified within the ER group. Taken together, results examining relative and absolute changes in outcomes from 3 months to 6 months underscore 2 phenomena. First, the pattern of long-term influence on outcomes exerted by 2 treatments, differing quite dramatically in duration and intensity, were similar. Second, a one-time skills-based group psychosocial treatment for chronic pain can lead to sustained improvements across a breadth of outcomes.

Clinical implications of our results are many. First, that 2-hour ER showed long-term outcomes comparable to 16-hour CBT hints that ER may represent a viable and practical alternative to CBT for some patients. However, concluding from these results that ER could outright replace longer-course CBT would be erroneous. The appropriate context of use for ER must be evaluated with a study across different demographic and pain disorder samples. The results do support ER's value as a rapid, scalable, and effective behavioral pain treatment that provides clinically meaningful improvements with less provider and patient time and cost. Second, one critical difference between ER and CBT is that the latter includes ongoing instructor-led and peer-supported practice of cognitive and behavioral skills to change how patients manage their pain. Our finding that ER and CBT have similar effects on outcomes raises a few possibilities. First, although ongoing group skills training and group practice may not be entirely necessary to achieve satisfactory outcomes, it is also plausible that group CBT dilutes the key mechanisms of change by including a breadth of content and skills that may or may not be relevant to individual patients. Teaching people about mind–body connections in chronic pain and the importance of thoughts, emotions, and behavior in governing responses to pain and stress, conveyed by ER in a single 2-hour didactic session, may be sufficient to produce meaningful results. Recent findings provide a context for understanding the results of this study. Thorn et al.^36^ and Williams et al.^42^ found that well-crafted pain education interventions achieved effects on outcomes statistically equivalent to those showed by active treatment comparators including CBT and mindfulness meditation. Similar to pain education interventions, ER does not provide ongoing *therapist-guided* skills practice.

Taken together, our findings—and those reporting similar effects for pain education interventions—suggest that a critical ingredient of psychosocial pain interventions may be imparting comprehensive and comprehensible information about the nature of chronic pain and key self-regulatory skills that enhance a person's ability to cope with and manage it. Third, we focused only on mean responses to the treatments, and did not take into account or analyze the variability around these means. Systematic study of variability in treatment responses and the factors related to this variability may pave the way for identifying which patients could benefit from ER and which patients would be better served by participating in the full course of group CBT. Uncovering moderators or predictors of differential response to ER vs CBT could provide the basis for decision-making guidelines regarding who would need multisession therapist-supported skills training and practice of CBT necessary for optimal outcomes, and for whom 1-session ER would be sufficient for achieving optimal outcomes.

Caveats must be issued. First, as reported previously,^13^ the pretreatment attrition rate was greatest in the HE group despite treatment expectations being similar across treatment groups. We minimized the impact of pretreatment attrition with intention-to-treat analyses. Moreover, we highlight that 6-month posttreatment attrition was similar between the ER and CBT groups, the primary focus of the current report. Second, we conducted the study at a single site in the San Francisco Bay Area of the United States. In factors that may limit generalizability of study findings, the sample required chronic low back pain and primarily consisted of white, college-educated participants; moreover, psychological comorbidities for mood and anxiety disorders—formally diagnosed using semistructured interviews—were notably low in this sample. As such, the study findings may not generalize to other pain conditions, diverse populations, or individually delivered CBT. We highlight that nearly half of the study sample had a chronic pain comorbidity in addition to the requisite low back pain (total of 2 pain conditions), and about one-fifth of the sample had 3 or more pain conditions.

Our findings in this work add to those of recent studies featuring ER. The feasibility and 3-month posttreatment efficacy of online-delivered ER in clinic patients with mixed-etiology chronic pain was recently reported.^29^ Moreover, 2 randomized trials have tested a fully-automated and tailored version of ER in 2 surgical populations with results showing clinically important benefits for reduced opioid use in breast cancer surgery^33^ and reductions in postsurgical pain upto 3 months after orthopedic trauma surgery.^34^ Furthermore, a current effectiveness trial is comparing an online 1-session ER to an online 8-session group CBT in a national sample of 1200 diverse adult patients (in race, ethnicity, and socioeconomic status) with a variety of chronic pain conditions in the U.S.^35^ Beyond treatment efficacy are questions about what are the necessary elements of psychosocial treatments to achieve satisfactory outcomes for different people. If further research in diverse samples finds the efficacy of single-session ER compared with group CBT and other interventions (eg, Mindfulness Based Stress Reduction and Acceptance and Commitment Therapy), dissemination and implementation of ER would have substantial clinical implications for underserved populations and under-resourced settings. Because of its low burden and cost, ER, especially when delivered online, could help dismantle many barriers to effective, accessible, and holistic chronic pain care in the U.S. and beyond.

## Disclosures

Stanford University receives revenue for continuing medical education on Empowered Relief (ER) instructor certification training provided to clinicians. Dr. Darnall is Chief Science Advisor at AppliedVR, and her consulting role with this company (personal fees) is unrelated to the current research. Dr. Darnall receives royalties for 4 pain treatment books she has authored or coauthored. She is the principal investigator for 2 pain research awards from the Patient-Centered Research Outcomes Research Institute, both of which involve either E.R., C.B.T., or both. Dr. Darnall is principal investigator for 2 NIH grants that are investigating the efficacy of E.R. Dr. Darnall serves on the Board of Directors for the American Academy of Pain Medicine, the Board of Directors for the Institute for Brain Potential, and the Medical Advisory Board for the Facial Pain Association. Dr. Darnall is a scientific member of the NIH Interagency Pain Research Coordinating Committee, a former member of the Centers for Disease Control and Prevention Opioid Workgroup (2020–2021), and a current member of the Pain Advisory Group of the American Psychological Association. Dr. Mackey receives research funding from the NIH, Food and Drug Administration, and Patient-Centered Outcomes Research Institute (administered through Stanford University). He is an unpaid advisor to both ACTTION (Analgesic, Anesthetic, and Addiction Clinical Trial Translations, Innovations, Opportunities, and Networks) on their oversight committee, and the American Chronic Pain Association (ACPA) for their scientific oversight. All other authors report no disclosures or conflicts of interest.

## Appendix A. Supplemental digital content

Supplemental digital content associated with this article can be found online at http://links.lww.com/PR9/A213.
